# Supplementary material for: Benchmarking phasing software with a whole-genome sequenced cattle pedigree
Source: BMC Genomics. 2022 Feb 15;23:130. doi: 10.1186/s12864-022-08354-6 (PMC8845340; doi:10.1186/s12864-022-08354-6)
Supplement: Supplementary file 1 — Additional file 1: Figure S1. Switch error rates in earlier versions of evaluated software in both scenarios. Boxplots of switch error rates (SER, %) obtained with AlphaPhase1.1, AlphaPhase1.3, Beagle3.3, Beagle4.0, Beagle4.1, Beagle5.0, Beagle5.1, Beagle5.2, ShapeIT2 and ShapeIT4.1, computed for the 98 validation individuals (A) in scenario 1 with only the 98 validation individuals, and (B) in scenario 2 with the 264 sequenced individuals. Figure S2. Haplotype block length metrics in earlier versions of evaluated software in both scenarios. (A, B) Quality adjusted (QA) haplotype block lengths (Mb), and (C, D) number of SNPs in correctly phased blocks (× 1000), obtained with AlphaPhase1.1, AlphaPhase1.3, Beagle3.3, Beagle4.0, Beagle4.1, Beagle5.0, Beagle5.1, Beagle5.2, ShapeIT2 and ShapeIT4.1, computed for the 98 validation individuals and plotted as a function of the proportion of the genome, in scenario 1 (A, C) with only the 98 validation individuals, and in scenario 2 (B, D) with the 264 sequenced individuals. The black curves with dots represent the total length of each chromosome (if they were perfectly phased) as a function of the proportion of the genome they cover. Figure S3. Distribution of haplotype block lengths in both scenarios. The quality adjusted (QA) haplotype block lengths (Mb) were obtained with AlphaPhase1.3, Beagle4.1, Beagle5.2, Eagle2.4, FImpute3.0 and ShapeIT4.1, computed for the 98 validation individuals (A) in scenario 1 with only the 98 validation individuals, and (B) in scenario 2 with the 264 sequenced individuals. Red stars represent mean values. Figure S4. Pedigree tree of the 264 Holstein-Friesian cattle used in this study. Individuals in blue are males, the ones in orange are females, and the ones surrounded in red are the 98 validation individuals (offspring from trios, that is with both parent sequenced) used to compare the algorithms. Table S1. Number of SNPs per chromosome in both scenarios. Total number of whole-genome sequence [file 12864_2022_8354_MOESM1_ESM.pdf]

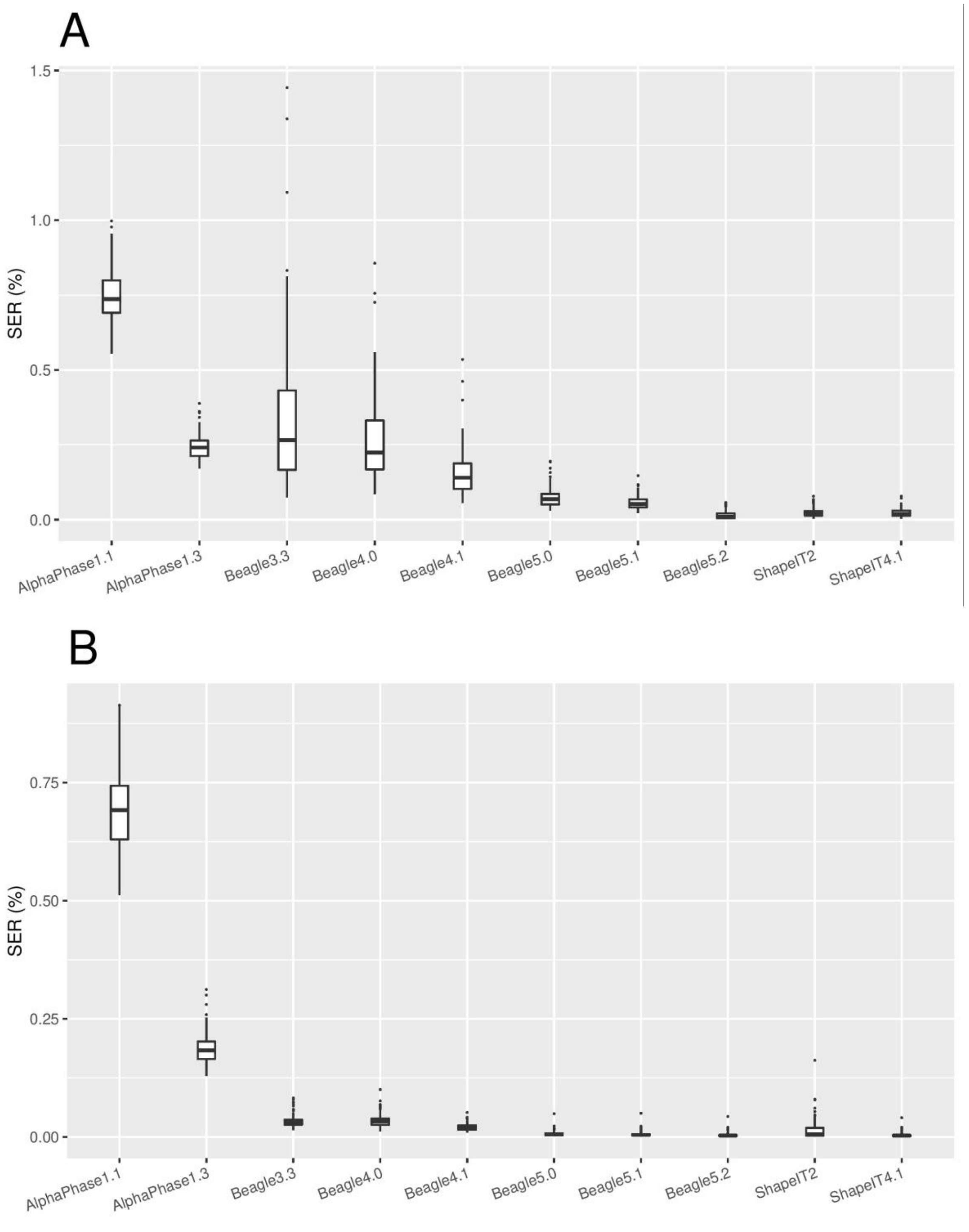

Figure S1. Switch error rates in earlier versions of evaluated software in both scenarios. *Boxplots of switch error rates (SER, %) obtained with AlphaPhase1.1, AlphaPhase1.3, Beagle3.3, Beagle4.0, Beagle4.1, Beagle5.0, Beagle5.1, Beagle5.2, ShapeIT2 and ShapeIT4.1, computed for the 98 validation individuals (A) in scenario 1 with only the 98 validation individuals, and (B) in scenario 2 with the 264 sequenced individuals.*

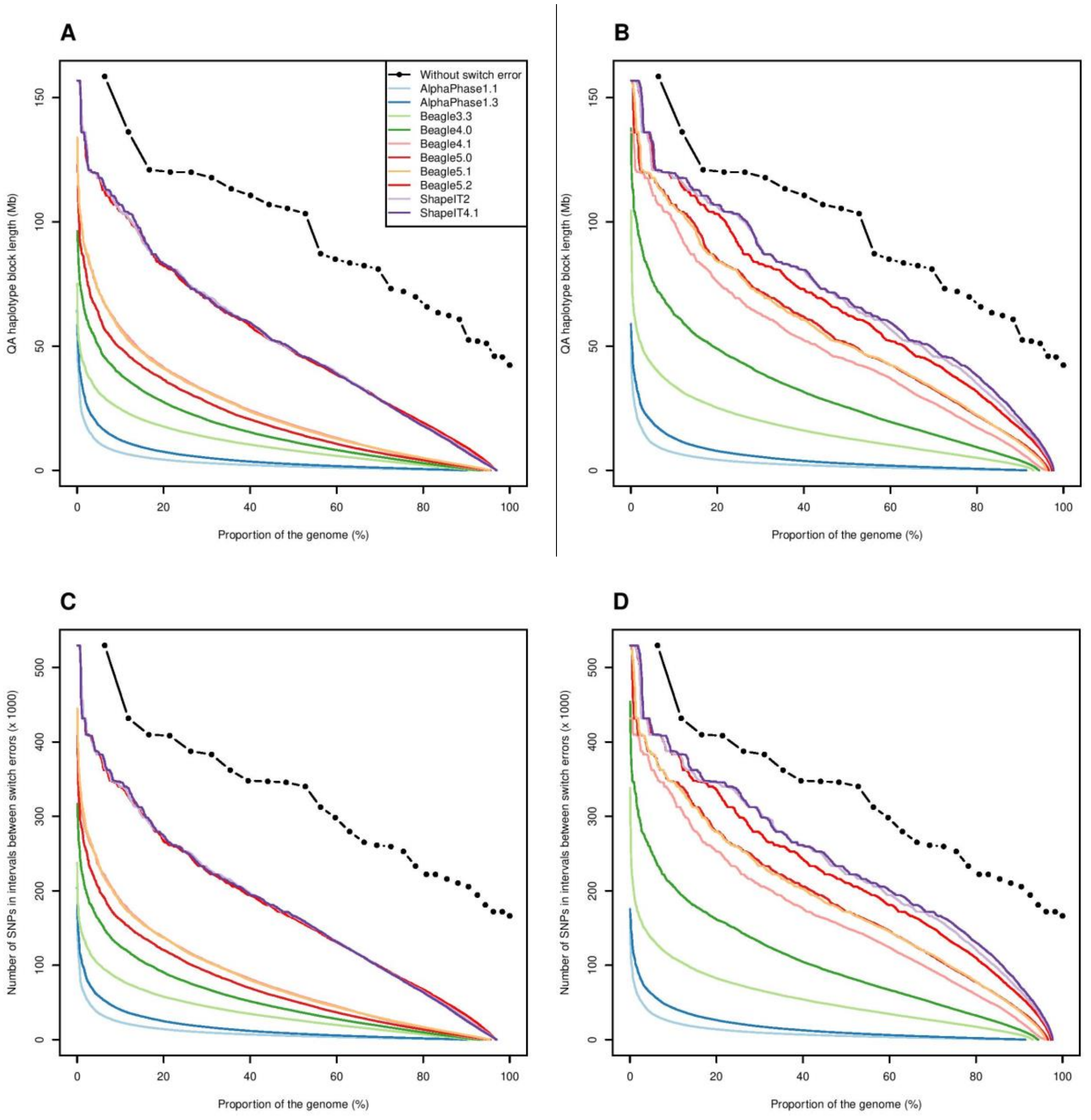

Figure S2. Haplotype block length metrics in earlier versions of evaluated software in both scenarios. *(A, B) Quality adjusted (QA) haplotype block lengths (Mb), and (C, D) number of SNPs in correctly phased blocks (x 1000), obtained with AlphaPhase1.1, AlphaPhase1.3, Beagle3.3, Beagle4.0, Beagle4.1, Beagle5.0, Beagle5.1, Beagle5.2, ShapeIT2 and ShapeIT4.1, computed for the 98 validation individuals and plotted as a function of the proportion of the genome, in scenario 1 (A, C) with only the 98 validation individuals, and in scenario 2 (B, D) with the 264 sequenced individuals. The black curves with dots represent the total length of each chromosome (if they were perfectly phased) as a function of the proportion of the genome they cover.*

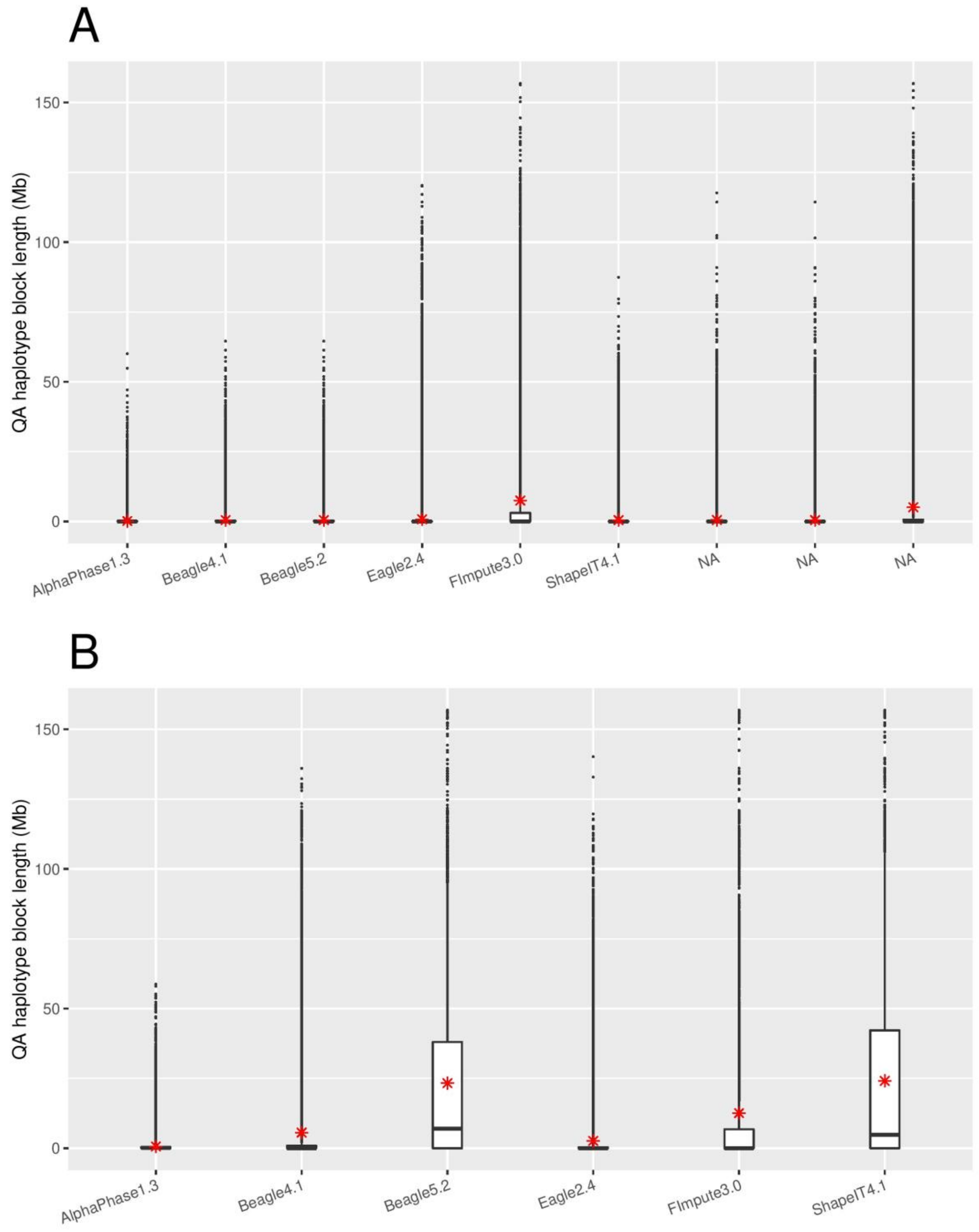

Figure S3. Distribution of haplotype block lengths in both scenarios. *The quality adjusted (QA) haplotype block lengths (Mb) were obtained with AlphaPhase1.3, Beagle4.1, Beagle5.2, Eagle2.4, FImpute3.0 and ShapeIT4.1, computed for the 98 validation individuals (A) in scenario 1 with only the 98 validation individuals, and (B) in scenario 2 with the 264 sequenced individuals. Red stars represent mean values.*

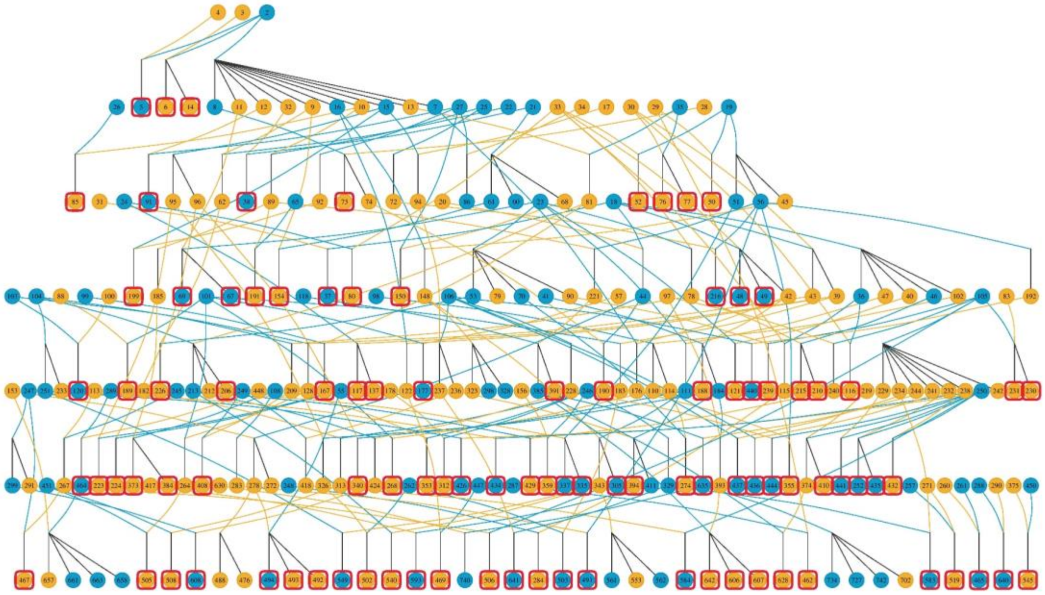

Figure S4. Pedigree tree of the 264 Holstein-Friesian cattle used in this study. *Individuals in blue are males, the ones in orange are females, and the ones surrounded in red are the 98 validation individuals (offspring from trios, that is with both parent sequenced) used to compare the algorithms.*

Chromosome	Number of SNPs after applying filtering procedures (see *Methods*)	Number of SNPs after removing monomorphic SNPs and SNPs with a missing genotyping rate below 5%		Mean number of heterozygous SNPs across the 98 offspring	
		Scenario 1	Scenario 2	Scenario 1	Scenario 2
1	529,626	529,582	529,600	121,003	121,002
2	431,779	431,702	431,719	102,524	102,524
3	387,566	387,531	387,547	88,918	88,918
4	408,467	408,433	408,446	96,956	96,956
5	383,157	383,127	383,146	89,821	89,822
6	409,796	409,756	409,785	98,721	98,721
7	340,141	340,070	340,126	79,742	79,742
8	346,970	346,941	346,964	79,739	79,739
9	345,865	345,644	345,847	79,126	79,126
10	347,854	347,839	347,844	79,916	79,915
11	362,191	362,170	362,179	84,009	84,009
12	312,506	312,477	312,491	73,220	73,221
13	265,193	265,167	265,183	61,133	61,133
14	279,542	279,528	279,534	64,147	64,147
15	298,393	298,364	298,372	69,585	69,586
16	259,508	259,454	259,497	61,050	61,050
17	261,305	261,292	261,300	62,710	62,710
18	215,908	215,900	215,904	50,969	50,969
19	210,452	210,447	210,450	49,474	49,474
20	252,881	252,860	252,875	59,498	59,498
21	233,326	233,248	233,308	54,239	54,239
22	205,619	205,611	205,617	48,671	48,671
23	222,026	222,000	222,018	51,325	51,326
24	222,142	222,129	222,134	51,827	51,827
25	166,292	166,279	166,286	39,031	39,031
26	171,810	171,803	171,799	38,886	38,884
27	181,041	181,030	181,034	43,365	43,365
28	172,002	171,992	171,997	38,908	38,908
29	194,321	194,306	194,318	45,708	45,709
Total	8,417,679	8,416,682	8,417,320	1,964,220	1,964,220

Table S1. Number of SNPs per chromosome in both scenarios. *Total number of whole-genome sequence SNPs and mean numbers of informative SNPs for each chromosome in each scenario (scenario 1: using the 98 validation individuals; scenario 2: using the 264 sequenced individuals).*

Metric	Min. SEC		Mean SEC		Median SEC		Max. SEC	
Scenario	1	2	1	2	1	2	1	2
AlphaPhase1.1	10,790	10,338	14,767	13,763	14,341	13,439	19,930	18,982
AlphaPhase1.3	3,339	2,635	4,796	3,698	4,612	3,548	7,675	6,169
Beagle3.3	1,452	289	6,595	643	5,209	600	28,854	1,596
Beagle4.0	1,655	241	5,121	657	4,435	648	16,925	1,940
Beagle4.1	1,032	196	3,113	399	2,769	359	10,578	999
Beagle5.0	595	30	1,491	128	1,339	98	3,960	949
Beagle5.1	455	26	1,107	108	1,024	81	2,913	966
Beagle5.2	35	13	293	75	191	55	1,136	834
Eagle2.4	2,799	325	5,036	894	4,901	882	9,031	1,535
FImpute3.0	3,012	2	4,603	165	4,477	115	7,616	598
ShapeIT2	64	8	459	307	377	110	1,551	3,277
ShapeIT4.1	64	12	449	72	386	50	1,561	785

Table S2. Summary statistics of the switch error counts in both scenarios. *Minimum, mean, median and maximum switch error counts (SEC), obtained with all the evaluated LD-based phasing algorithms, computed for the 98 validation individuals in each scenario (scenario 1: using the 98 validation individuals; scenario 2: using the 264 sequenced individuals).*

Metric	Min. SER (%)		Mean SER (%)		Median SER (%)		Max. SER (%)	
Scenario	1	2	1	2	1	2	1	2
AlphaPhase1.1	0.5547	0.5120	0.7510	0.7000	0.7369	0.6919	0.9977	0.9139
AlphaPhase1.3	0.1716	0.1293	0.2440	0.1882	0.2412	0.1834	0.3884	0.3122
Beagle3.3	0.0746	0.0145	0.3366	0.0328	0.2657	0.0300	1.4426	0.0825
Beagle4.0	0.0851	0.0121	0.2609	0.0336	0.2240	0.0322	0.8564	0.1003
Beagle4.1	0.0556	0.0091	0.1583	0.0204	0.1404	0.0183	0.5353	0.0517
Beagle5.0	0.0306	0.0015	0.0756	0.0066	0.0688	0.0050	0.1954	0.0491
Beagle5.1	0.0227	0.0013	0.0564	0.0056	0.0523	0.0041	0.1474	0.0500
Beagle5.2	0.0018	0.0007	0.0150	0.0039	0.0093	0.0027	0.0575	0.0431
Eagle2.4	0.1439	0.0167	0.2561	0.0455	0.2485	0.0451	0.4570	0.0753
FImpute3.0	0.1371	0.0001	0.2343	0.0085	0.2256	0.0059	0.3854	0.0316
ShapeIT2	0.0032	0.0004	0.0234	0.0157	0.0187	0.0056	0.0785	0.1620
ShapeIT4.1	0.0032	0.0006	0.0229	0.0037	0.0192	0.0026	0.0790	0.0406

Table S3. Summary statistics of the switch error rates in both scenarios. *Minimum, mean, median and maximum switch error rates (SER, %), obtained with all the evaluated LD-based phasing algorithms, computed for the 98 validation individuals in each scenario (scenario 1: using the 98 validation individuals; scenario 2: using the 264 sequenced individuals).*

Metric	Min. QA haplotype block length (bp)		Mean QA haplotype block length (bp)		Median QA haplotype block length (bp)		Max. QA haplotype block length (bp)	
Scenario	1	2	1	2	1	2	1	2
AlphaPhase1.1	1	1	152,648	163,711	621	725	58,851,352	59,132,672
AlphaPhase1.3	1	1	465,447	610,536	10,653	55,973	64,541,591	58,753,065
Beagle3.3	1	1	346,928	3,446,510	166	13,201	75,174,431	104,596,496
Beagle4.0	1	1	453,121	3,426,126	337	1,893	96,286,002	137,647,941
Beagle4.1	1	1	754,558	5,595,676	484	45,729	120,319,442	136,047,557
Beagle5.0	1	1	1,564,542	15,344,484	9,118	1,898,160	124,880,023	156,851,007
Beagle5.1	1	1	2,090,531	17,464,973	7,924	4,271,136	133,971,707	156,846,286
Beagle5.2	1	1	7,479,255	23,313,031	12,334	6,953,676	156,851,371	156,851,371
Eagle2.4	1	1	471,275	2,618,868	172	161	87,438,003	140,190,109
FImpute3.0	1	1	501,289	12,561,540	583	27,490	117,669,102	156,851,371
ShapeIT2	1	1	4,950,799	7,244,068	1,456	433	156,851,371	156,851,371
ShapeIT4.1	1	1	5,050,285	24,108,523	2,134	4,738,542	156,851,371	156,851,371

Table S4. Summary statistics of the quality adjusted haplotype block lengths in both scenarios. *Minimum, mean, median and maximum quality adjusted (QA) haplotype block lengths, obtained with all the evaluated LD-based phasing algorithms, computed for the 98 validation individuals in each scenario (scenario 1: using the 98 validation individuals; scenario 2: using the 264 sequenced individuals).*

Metric	Median SEC		Median SER (%)		Median QA haplotype block length (bp)		QAN50 (bp)	
Scenario	1	2	1	2	1	2	1	2
AlphaPhase1.3	12,627	9,343	0.6483	0.4802	2,089	2,015	682,661	423,410
Beagle4.1	4,269	2,316	0.2179	0.1168	954	4,735	3,566,015	5,142,027
Beagle5.2	2,486	1,965	0.1247	0.1060	9,123	9,378	5,008,038	5,949,264
Eagle2.4	6,965	2,676	0.3511	0.1373	223	547	2,514,025	4,907,412
FImpute3.0	19,440	6,055	0.9994	0.3150	741	741	1,057,215	4,089,255
ShapeIT4.1	2,429	1,911	0.1229	0.1028	3,961	3,442	5,438,297	6,561,661

Table S5. Results of different metrics used to assess phasing quality in both scenarios before improving genotype quality with Beagle4.1. *Median values of switch error counts (SEC), switch error rates (SER, %), quality adjusted (QA) haplotype block length (bp), and QAN50 (bp), obtained with AlphaPhase1.3, Beagle4.1, Beagle5.2, Eagle2.4, FImpute3.0 and ShapeIT4.1, computed for the 98 validation individuals in each scenario (scenario 1: using the 98 validation individuals; scenario 2: using the 264 sequenced individuals).*

QA haplotype block length (Mb)	1		5		10		50	
Scenario	1	2	1	2	1	2	1	2
AlphaPhase1.3	40.5	33.7	6.4	5.5	2.0	1.7	0.0	0.0
Beagle4.1	79.2	84.4	40.0	50.8	19.8	29.7	0.2	0.9
Beagle5.2	83.6	85.9	50.0	55.0	29.5	34.1	0.9	1.8
Eagle2.4	74.7	85.2	28.8	49.4	11.7	27.4	0.2	0.4
FImpute3.0	51.6	79.4	11.1	44.7	3.6	28.1	0.0	8.8
ShapeIT4.1	84.5	87.1	52.2	57.6	31.9	37.3	1.4	2.6

Table S6. Genome percentage included in correctly phased segments longer than different thresholds in both scenarios before improving genotype quality with Beagle4.1. *Percentage of the genome covering quality adjusted (QA) haplotype blocks of minimal length of respectively 1, 5, 10 and 50 Mb, obtained with AlphaPhase1.3, Beagle4.1, Beagle5.2, Eagle2.4, FImpute3.0 and ShapeIT4.1, computed for the 98 validation individuals in each scenario (scenario 1: using the 98 validation individuals; scenario 2: using the 264 sequenced individuals).*

Metric	Median SEC	Median SER (%)	Median QA haplotype block length (bp)	QAN50 (bp)
Scenario	2	2	2	2
`--pbwt-depth` 4 (default)	50.0	0.0026	4,738,542	69,002,979
`--pbwt-depth` 8	50.0	0.0026	4,907,992	69,331,250
`--pbwt-depth` 16	49.5	0.0026	4,644,078	69,347,954
`--pbwt-depth` 32	49.0	0.0026	4,613,477	69,348,191
`--pbwt-depth` 64	50.0	0.0026	4,264,849	69,350,909
`--pbwt-depth` 128	49.5	0.0026	3,950,800	69,350,672

Table S7. Results of different metrics used to assess phasing quality in the second scenario using different parameter settings with ShapeIT4.1. *Median values of switch error counts (SEC), switch error rates (SER, %), quality adjusted (QA) haplotype block length (bp), and QAN50 (bp), obtained with ShapeIT4.1 using different values (4, 8, 16, 32, 64 and 128) of the parameter `--pbwt-depth`, computed for the 98 validation individuals (scenario 2: using the 264 sequenced individuals).*

QA haplotype block length (Mb)	1	5	10	50
Scenario	2	2	2	2
`--pbwt-depth` 4 (default)	97.64	96.76	95.07	69.21
`--pbwt-depth` 8	97.69	96.85	95.07	70.17
`--pbwt-depth` 16	97.74	96.87	95.14	70.51
`--pbwt-depth` 32	97.67	96.82	95.12	70.29
`--pbwt-depth` 64	97.69	96.83	95.11	90.46
`--pbwt-depth` 128	97.75	96.89	95.17	70.25

Table S8. Genome percentage included in correctly phased segments longer than different thresholds in the second scenario using different parameter settings with ShapeIT4.1. *Percentage of the genome covering quality adjusted (QA) haplotype blocks of minimal length of respectively 1, 5, 10 and 50 Mb, obtained with ShapeIT4.1 using different values (4, 8, 16, 32, 64 and 128) of the parameter `--pbwt-depth`, computed for the 98 validation individuals (scenario 2: using the 264 sequenced individuals).*

Metric	Median SEC	Median SER (%)	Median QA haplotype block length (bp)	QAN50 (bp)
Scenario	1	1	1	1
AlphaPhase1.1	14,341	0.7369	621	1,540,038
AlphaPhase1.1-ped	15,884	0.8276	1,721	848,214
FImpute3.0	4,477	0.2256	583	6,114,640
FImpute3.0-ped	4,715	0.2426	562	5,772,640

Table S9. Results of different metrics used to assess phasing quality in the first scenario without and with the pedigree information. *Median values of switch error counts (SEC), switch error rates (SER, %), quality adjusted (QA) haplotype block length (bp), and QAN50 (bp), obtained with AlphaPhase1.1 and FImpute3.0 without and with (-ped) the pedigree information, computed for the 98 validation individuals (scenario 1: using the 98 validation individuals).*

QA haplotype block length (Mb)	1	5	10	50
Scenario	1	1	1	1
AlphaPhase1.1	61.73	16.64	5.93	0.02
AlphaPhase1.1-ped	45.86	9.92	3.27	0.05
FImpute3.0	85.62	56.06	33.96	2.05
FImpute3.0-ped	85.05	54.44	31.68	1.74

Table S10. Genome percentage included in correctly phased segments longer than different thresholds in the first scenario without and with the pedigree information. *Percentage of the genome covering quality adjusted (QA) haplotype blocks of minimal length of respectively 1, 5, 10 and 50 Mb, obtained with AlphaPhase1.1 and FImpute3.0 without and with (-ped) the pedigree information, computed for the 98 validation individuals (scenario 1: using the 98 validation individuals).*
